# The impact of non-invasive manual and ultrasonographic reduction for incarcerated obturator hernia: a retrospective cohort study and systematic review

**DOI:** 10.1007/s10029-024-03119-4

**Published:** 2024-07-29

**Authors:** Fuyumi Kobayashi, Jun Watanabe, Masaru Koizumi, Hironori Yamaguchi, Naohiro Sata

**Affiliations:** 1 Department of Surgery, Division of Gastroenterological, General and Transplant Surgery, Jichi Medical University, Shimotsuke-City, Tochigi Japan; 2 Department of Surgery, Tochigi Medical Center Shimotsuga, Tochigi-City, Tochigi Japan; 3 Division of Community and Family Medicine, Jichi Medical University, 3311-1 Yakushiji, Shimotsuke-City, Tochigi 329-0498 Japan

**Keywords:** Incarcerated obturator hernia, Ultrasonography reduction, Manual reduction, Systematic review

## Abstract

**Purpose:**

Non-invasive reduction in patients with incarcerated obturator hernias is an emergency surgery alternative. There are two non-invasive reduction types: manual and ultrasonographic (ultrasound-guided and ultrasound-assisted reduction). However, the impact of ultrasound guidance on manual reduction has not been adequately evaluated. We aimed to compare non-invasive ultrasound reduction with manual reduction in patients with incarcerated obturator hernias.

**Methods:**

We searched MEDLINE, Cochrane Central Library, Embase, Ichushi Web, ClinicalTrial.gov, and ICTRP for relevant studies. The primary outcomes were success and bowel resection rates. We performed a subgroup analysis between ultrasound-guided and ultrasound-assisted reductions. This study was registered in PROSPERO (CRD 42,024,498,295).

**Results:**

We included six studies (112 patients, including 12 from our cohort). The success rate was 78% (69 of 88 cases) with ultrasonographic reduction and 33% (8 of 24 cases) with manual reduction. The success rate was higher with ultrasonographic than with manual reduction. Subgroup analysis revealed no significant difference between ultrasonography-assisted (76%) and ultrasonography-guided (80%) reductions (*p* = 0.60). Non-invasive reductions were predominantly successful within 72 h of onset, although durations extended up to 216 h in one case. Among the successful reduction cases, emergency surgery and bowel resection were necessary in two cases after 72 h from onset. Bowel resection was required in 48% (12 of 25), where the non-invasive reduction was unsuccessful within 72 h of confirmed onset.

**Conclusions:**

Ultrasonographic reduction can be a primary treatment option for patients with obturator hernias within 72 h of onset by emergency physicians and surgeons on call. Future prospective studies are needed to evaluate ultrasonographic reduction’s impact.

**Supplementary Information:**

The online version contains supplementary material available at 10.1007/s10029-024-03119-4.

## Introduction

Obturator hernia, a rare but significant clinical condition, accounts for approximately 0.08–1% of all abdominal hernias [1]. Primarily affecting thin, older women, these hernias often lead to emergency small bowel obstructions [2]. The diagnosis of obturator hernias is particularly challenging due to their non-specific symptoms and their location, rendering them unsuitable for direct examination and palpation [2]. Consequently, the diagnosis and subsequent intervention are frequently delayed, leading to a high rate of small bowel resection of almost 40% [2]. Complicated obturator hernias are associated with a mortality rate ranging from 10 to 28%, largely attributed to the advanced age and significant comorbidities of patients [2–4]. Therefore, prompt diagnosis and emergency surgery are important in the treatment of incarcerated obturator hernias [5].

**Fig. 1:**
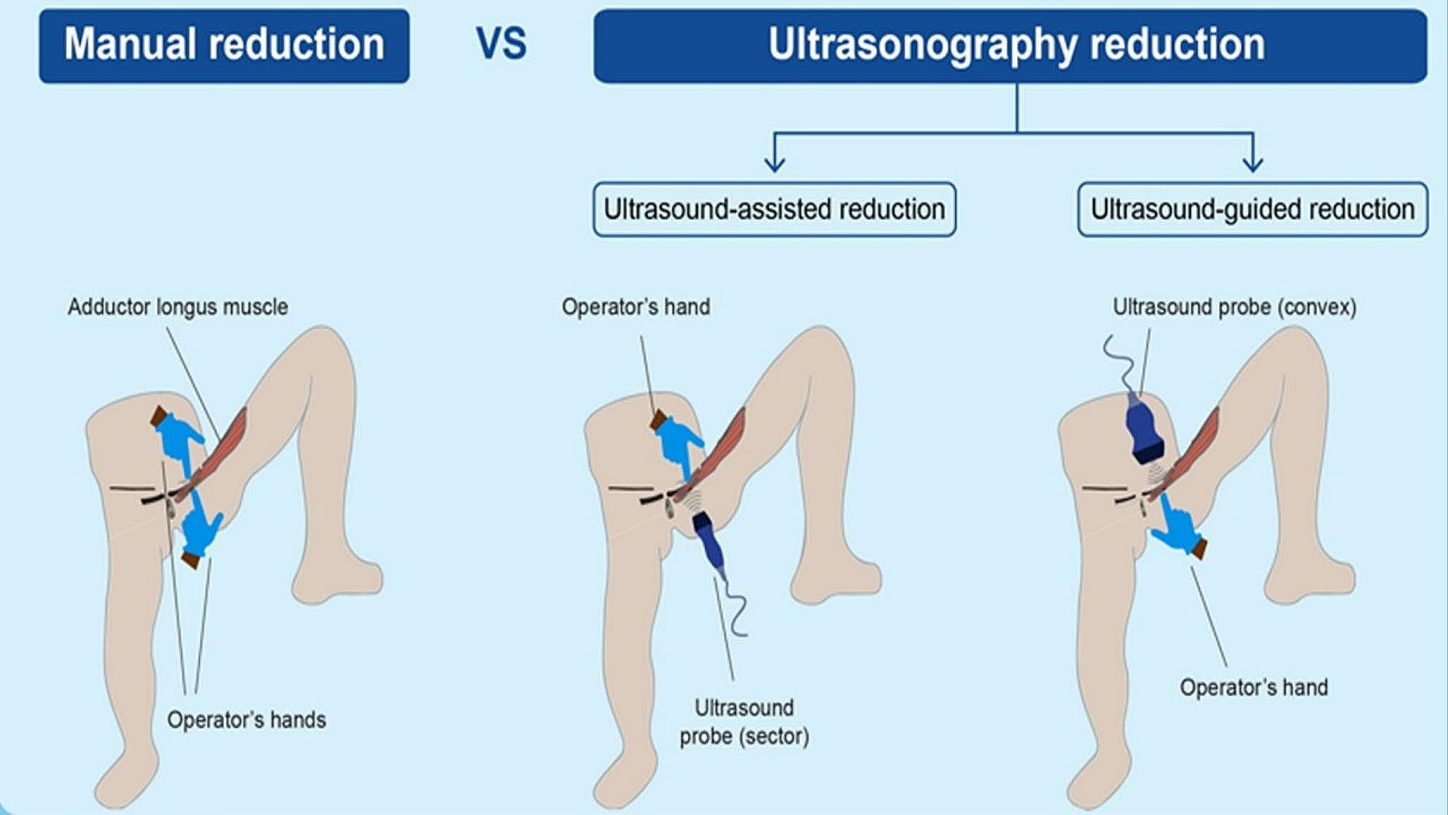
Ultrasound-assisted and ultrasound-guided reduction methods

Manual reduction is a non-invasive approach that has been explored as an alternative to emergency surgery for the management of obturator hernias [6, 7]. In recent years, non-invasive methods of ultrasound (US) reduction for obturator hernias have been developed (Fig. 1). One method is US-guided reduction, during which the surgeon holds a convex- or linear-type probe on the patient’s anterior thigh, between the femoral vein and long adductor muscle, using the left hand while pressing on the hernia sac with the right hand. Another method is US-assisted reduction, which involves the surgeon applying pressure in the same thigh area with their left hand while pressing on the hernia sac using a sector-type echo probe with their right hand. This method enables direct compression and visual monitoring of the trapped intestinal segment within the hernial sac on the dorsal side. With both methods, the patient is positioned on their back with their hips and knees bent. To aid in repositioning, an assistant may gently rotate the hip joint laterally and repeatedly open the hernial orifice, as well as extend the hip and knee joints [8, 9]. Although these techniques have shown success in various case reports, their success rate has not been fully evaluated because of the limited number of cases [10, 11].

This gap in clinical knowledge underscores the important need for more comprehensive data on the success rates of non-invasive reduction of obturator hernias. Therefore, in this review, we aimed to compare the impact of non-invasive US reduction with that of manual reduction in patients with incarcerated obturator hernias.

## Methods

### Non-invasive reduction for incarcerated obturator hernias

There are two non-invasive reduction types: manual and ultrasonographic (ultrasound-guided and ultrasound-assisted reduction).

### Manual reduction

The patient is positioned supine, with the affected leg placed in abduction, flexion, and external rotation. The surgeon uses one hand to apply pressure to the cephalic side of the adductor pollicis longus muscle. With the other hand, the surgeon follows along the posterior edge of the adductor pollicis longus muscle toward the pubis to locate the hernia site. Next, the surgeon identifies the slightly superior margin of the pubic tubercle externally and applies direct pressure to the hernia sac at the anterior aspect of the obturator foramen.

### Ultrasonographic reduction

#### Patient positioning

The patient is positioned supine, with the affected leg placed in abduction, flexion, and external rotation. The reduction technique requires two personnel. The assistant is responsible for gently and repeatedly moving the affected lower limb through abduction, flexion, and external rotation, followed by adduction, extension, and internal rotation of the hip joint.

#### US-Assisted reduction

The surgeon applies hand pressure to the cephalic side of the adductor longus muscle while positioning a sector-type ultrasound probe along the posterior (dorsal) edge of the adductor longus and gracilis muscles, directing it towards the pubis. This approach allows the identification of the advanced hernia site and reaches the slightly superior external margin of the pubic tubercle. Direct ultrasound pressure is then applied from the anterior aspect of the obturator foramen. There is a critical point where the assistant’s lower limb movements cause the internal and external obturator muscles to relax, facilitating the reduction of the incarcerated intestine. The real-time observation of the hernia reduction through the ultrasound probe, coupled with pressure applied using the sector-type ultrasound probe, prevents lateral displacement of the hernia sac, which is the key feature of this method. This technique is referred to as the modified Four hand Reduction for incarcerated Obturator hernia under Guidance of Sonograpy (FROGS) technique [8].

#### US-guided reduction

The surgeon places a convex ultrasound probe on the cephalic side of the adductor longus muscle, visually identifying the herniated intestine. The surgeon’s hand is then guided along the posterior (dorsal) edge of the adductor longus and gracilis muscles towards the pubis. This approach allows the identification of the slightly superior external margin of the pubic tubercle and access to the anterior aspect of the obturator foramen. The surgeon then manually applies pressure to the advanced hernia site. There is a critical point where the assistant’s lower limb movements cause the internal and external obturator muscles to relax, facilitating the reduction of the incarcerated intestine. The real-time observation of the reduction through the ultrasound probe, along with the tactile feedback transmitted to the surgeon’s fingertips, is the key feature of this method. This technique is commonly referred to as the original FROGS technique [8].

#### Confirmation of reduction

**Fig. 2:**
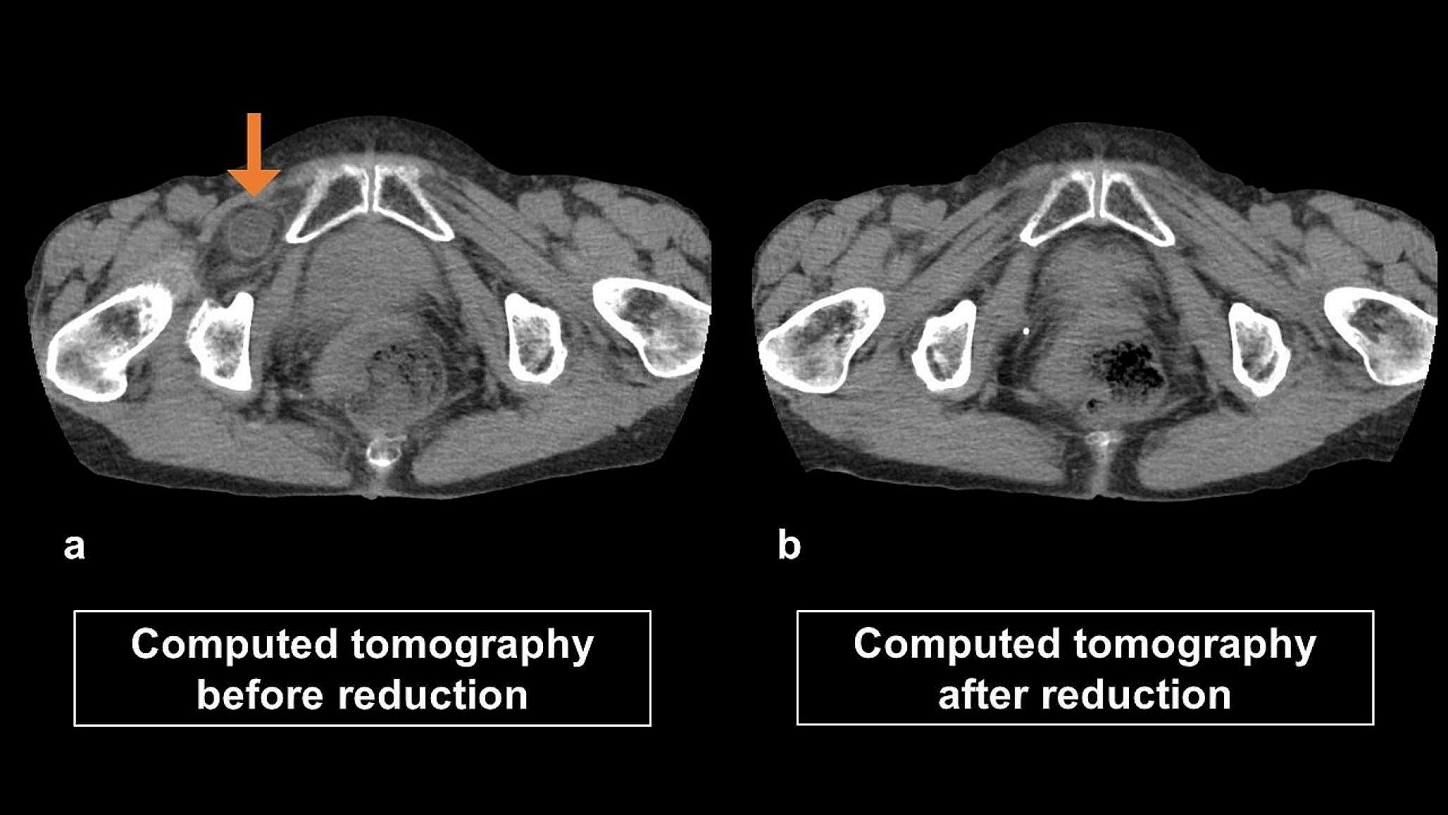
The computed tomography scan images before and after non-invasive reduction

Due to the difficulty in accurately determining the success of the reduction solely based on ultrasound findings, it is recommended to use computed tomography (CT) for confirmation (Fig. 2).

### Retrospective cohort study

In this single-center observational retrospective cohort study, we included patients with incarcerated obturator hernias treated at the study center between April 2016 and December 2023. We excluded patients with asymptomatic nonincarcerated obturator hernias (*n* = 3) and retrospectively reviewed patient characteristics, treatment methods, and surgical outcomes. The Institutional Review Board of the study center approved the study protocol, which complied with the provisions of the Declaration of Helsinki (Approval No. 216, approval year 2024). We obtained consent using an opt-out method. The first and corresponding authors take complete responsibility for the integrity of the data and the accuracy of the data analysis.

We diagnosed all patients with incarcerated obturator hernias using CT. Our non-invasive reduction included both manual and US reductions. Specifically, we used US-assisted reduction. Post-reduction, we used CT to evaluate the non-invasive reduction’s success. When non-invasive reduction was successful, we opted for an anterior approach or a transperitoneal or preperitoneal approach to surgery. When non-invasive reduction failed, we performed a lower midline laparotomy. We determined the necessity of bowel resection during surgery.

### Systematic review protocol

This study followed the Preferred Reporting Items for Systematic Reviews and Meta-Analysis (PRISMA) 2020 guidelines [12]. The protocol was registered in PROSPERO (CRD 42,024,498,295).

### Study selection

In this systematic review, we selected studies that assessed the effect of non-invasive obturator hernia reductions, including randomized controlled trials, cohort studies, and case-control studies. We excluded case series and case reports that did not include all cases during the study period and conference abstracts because success rates could not be calculated. We included adults aged ≥ 18 years in this systematic review, and the primary outcomes were the success rates of non-invasive reduction and bowel resection. The selection criteria for each reduction method were determined by each facility and the proficiency of each physician or surgeon.

We conducted extensive searches of several databases for studies published until January 4, 2024. These included the Cochrane Central Register of Controlled Trials (CENTRAL) via the Cochrane Library, MEDLINE via PubMed, and EMBASE via the ProQuest Dialog (Supplementary 1). We also searched the World Health Organization International Clinical Trials Platform Search Portal (ICTRP) and ClinicalTrials.gov for ongoing and unpublished trials (Supplementary 2). We examined the reference lists of all relevant studies, including international guidelines [13] and articles that cited these studies. Our inclusion criteria did not limit the inclusion of studies according to observation period, publication year, or country of origin. The main analysis included only English articles, but the sensitivity analysis included articles published in other languages as well. We contacted the authors of the original studies to acquire any unpublished or additional data.

### Data collection and analysis

Two reviewers (FK and JW) independently screened the titles and abstracts, followed by an assessment of eligibility based on the full text. The same two reviewers performed independent data extraction and evaluated the risk of bias using the Risk of Bias In Non-randomized Studies of Interventions tool [14]. Disagreements were resolved through discussion. If a consensus could not be reached, a third reviewer acted as an arbiter to make the final decision (MK).

### Statistical analysis

Continuous variables are expressed as medians and interquartile ranges (IQRs). Categorical variables are presented as numbers and percentages. We performed between-group comparisons using the Mann–Whitney U test for continuous and ordinal variables and the chi-squared test for categorical variables. No data were missing. The threshold for significance was set at *p* < 0.05.

For the main analysis, we simply calculated the overall number of successes and the total number of patients because the individual results for each study were known. To clarify the influence of effect modifiers on the results, we performed subgroup analyses of the success rates of US-guided and US-assisted reductions. We summarized bowel resection based on the definition in the original article; however, no meta-analysis of bowel resection has been performed. Additionally, we analyzed the patients separately before and 72 h after onset.

In this meta-analysis, we compared US reduction with manual reduction in studies that directly compared the two methods. We performed sensitivity analyses of the success rates to assess the robustness of the review results based on the decisions made during the review process; that is, the inclusion of studies assuming that the next case failed in a study with a 100% success rate, the exclusion of studies with a 100% success rate, and the inclusion of studies published in English or Japanese. We pooled the success rates of manual and US reductions, and the odds ratios (OR) and 95% confidence intervals (Cis) were used to compare the success rates of US reduction with manual reduction according to the Cochrane handbook guidelines [15]. We conducted an intention-to-treat analysis of the dichotomous data. We performed a meta-analysis using the data available in the original study using STATA SE16 software (version 16.1, Stata Corporation, College Station, TX, USA) with metaprop and metan using a random-effects model [16, 17]. We evaluated statistical heterogeneity by visually inspecting forest plots and calculating I^2^ (0–40%: might not be important; 30–60%: may represent moderate heterogeneity; 50–90%: may represent substantial heterogeneity; and 75–100%: considerable heterogeneity) based on the Cochrane handbook [15]. We performed an extensive literature search of unpublished trials using the Clinical Trial Registry System (ClinicalTrials.gov and ICTRP). We did not perform a funnel plot or Egger’s test because fewer than 10 eligible studies were included in the meta-analysis based on the Cochrane handbook guidelines [15].

## Results

### Retrospective cohort study

Twelve patients with obturator hernias were eligible for inclusion. The details of all cases are shown in Supplementary 3. The median age of the patients was 87 years (range 76–95 years). The median body mass index was 17.8 kg/m^2^ (range 15.2–22.0). Eight obturator hernias were observed on the patients’ right sides. The median time from symptom onset to reduction was 6 h (range 3–72 h).

Table 1 summarizes the characteristics of our patients with incarcerated obturator hernias in the US and manual reduction groups. Of 12 patients, US-assisted reduction was performed in eight patients, and manual reduction was performed in four patients. There were no significant differences in characteristics between the two groups.

**Table 1:** Summary of the characteristics, surgical treatment, and outcomes of our patients with incarcerated obturator hernias

	Ultrasound-assisted reduction	Manual reduction	*p*-value
Patients (male)	8 (0)	4 (0)	1.00
Age (years)	87 [82–93]	91 [87–94]	0.67
Body mass index (kg/m^2^)	17.8 [15.5–18.2]	17.1 [15.9–17.9]	0.93
Duration of symptoms (hours)	5 [3–24]	6 [3–72]	0.43
Lesion site (right)	6 (75)	2 (50)	0.39
Bedridden	2 (25)	1 (25)	0.39
Charlson comorbidity index	6 [5–7]	6 [6–7]	0.60
Dementia	4 (50)	1 (25)	0.41
Successful reduction	8 (100)	2 (50)	0.03
Patients who underwent emergency surgery	0 (0)	2 (50)	0.16
Bowel resection	0 (0)	2 (50)	0.03

BMI, body mass index; NR, not reported

All data are presented as median [interquartile range] and number (%)

The success rate for US reduction was 100% (8/8), whereas that for manual reduction was 50% (2/4) (*p* = 0.03). All patients who underwent US reduction also underwent elective surgery. Conversely, two patients who underwent manual reductions experienced failure and subsequently underwent emergency surgery, which necessitated intestinal resection (*p* = 0.03).

### Systematic review

#### Study selection

**Fig. 3:**
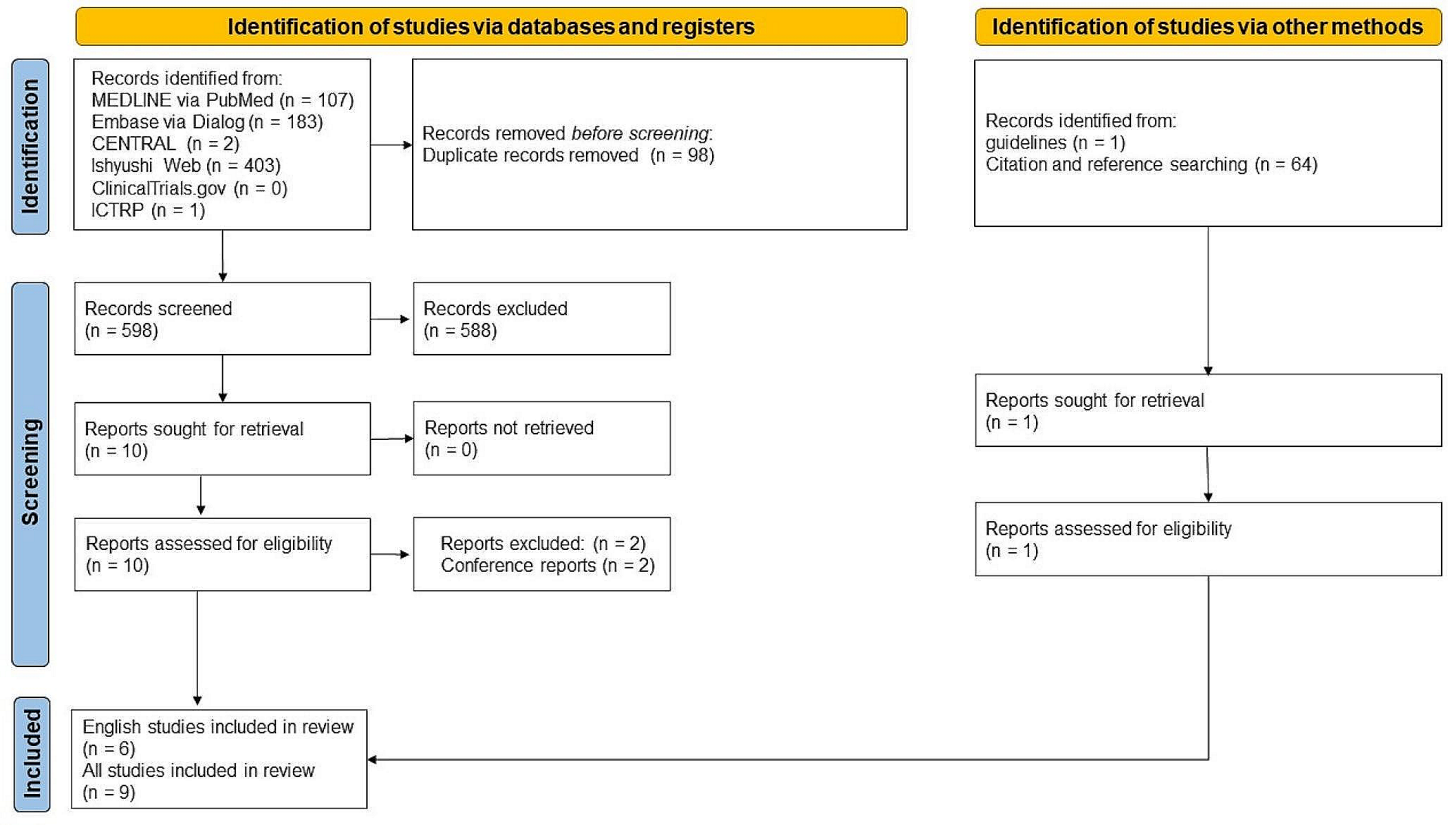
The process of study selection

Figure 3 illustrates the study selection process. A total of 696 records were identified from the six electronic databases as of January 4, 2024. Following a thorough screening process, nine studies encompassing 151 patients were ultimately selected for inclusion [6–9, 18–22].

Table 2 presents the characteristics of the included studies. All studies were published in Japan. Among these, six studies were published in English, and three were published in Japanese. The median age of the participants was 85 years (range 81–86 years). The median body mass index was reported as 17.4 kg/m^2^ (range 16.2–17.9). Additionally, the median time from symptom onset to symptom reduction was 23 h (range 1–96 h).

**Table 2:** Summary of the characteristics of the eligibility studies

Authors (ref no.)	Year	Country	Language	Subject (hernia) no.	Age (years)	BMI (kg/m^2^)	Reduction methods	Number of patients underwent reduction (success/total)	Time from onset to reduction in patients with successful reduction (hour)	Surgical methods in patients with successful reduction (laparoscopy/ anterior approach/ open/ no surgery)	Bowel resection cases with successful reduction (resection/total)	Surgical methods in patients with unsuccessful reduction (laparoscopy/ anterior approach/ open/ no surgery)	Bowel resection cases due to failure of reduction (resection/total)
Shigemitsu (7)	2012	Japan	English	5	81	17.9	Manual	4/5	12 (6–36)	4/0/0/0	0/4	NR	0/1
Mikami (18)	2012	Japan	Japanese	13	86	16.2	US-assisted	11/13	8 (1–48)	0/11/0/0	0/11	0/0/2/0	2/2
Tonai (19)	2015	Japan	Japanese/English	9	81	NR	Manual	1/1	96	0/1/0/0	0/1	0	0
							US-assisted	4/8	24 (12–24)	0/0/3/1	0/3	0/1/3/0	0/4
Kawanaka (9)	2018	Japan	English	9	NR	NR	US-assisted	6/9	NR	5/0/0/1	0/5	0/0/3/0	3/3
Hara (20)	2020	Japan	Japanese	10	85	17.5	US-guided	10/10	6 (2–72)	5/2/0/3	0/7	0	0
Maeda (21)	2021	Japan	English	12	85	17.4	US-assisted	10/12	NR	4/3/0/3	0/7	0/0/2/0	2/2
Togawa (8)	2022	Japan	Japanese/English	35	NR	NR	Manual	1/14	NR	1/0/0/0	0/1	NR	NR
							US-guided	20/20	23	6/0/0/14	0/6	0	0
Gokon (6)	2023	Japan	English	31	NR	NR	US-guided	21/31	16 cases within 72 h and 1 case at 216 h	(17)/2	2/2	NR	6/8

Supplementary 4 shows the risk of bias assessed using the Risk of Bias In Non-randomized Studies of Interventions tool. All overall risks of bias were critical because of bias due to confounding factors, such as the inability to adjust for time-dependent confounders and operator preferences.

#### Success rate of non-invasive reduction

The overall success rate was 69% (77 of 112 cases), with non-invasive reductions in studies published only in English. The success rate was 78% (69 of 88 cases) for US reduction and 33% (8 of 24 cases) for manual reduction (*p* = 0.46). There was no significant difference between US-associated (76%) and US-guided (80%) reductions (*p* = 0.60).

#### Bowel resection

Of the cases that were successfully reduced, 3% (2 of 77 cases) required emergency surgery and bowel resection. All patients in whom reduction failed required emergency surgery, and 58% (19 of 33) required bowel resections.

#### Patients before and after 72 h of onset

Non-invasive reduction was predominantly successful within 72 h of onset (74 of 77 cases), although the duration was up to 96 h in two cases and 216 h in one case. Cases that were successfully repaired within 72 h of onset did not require bowel resections. Only two patients with an onset-to-reduction time greater than 72 h required emergency surgery and bowel resections. Additionally, bowel resection was required in 48% (12 of 25) of the cases in which non-invasive reduction was not successful within 72 h of onset, and while 80% (4 of 5) of the cases in which non-invasive reduction was not successful later than 72 h after onset.

### Meta-analyses

**Fig. 4:**
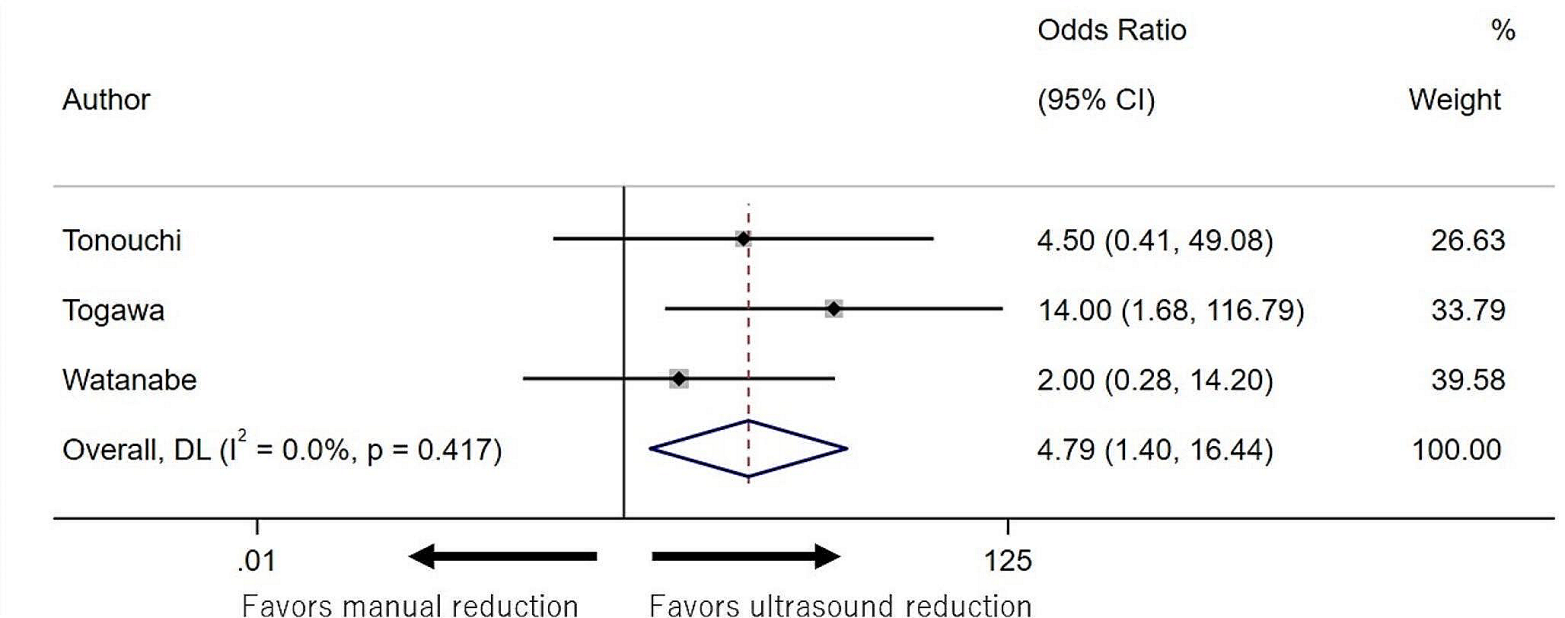
Forrest plot of the success rate in ultrasound reduction compared to manual reduction

In the meta-analysis of studies that directly compared the two methods, the success rate for US reduction was higher than that for manual reduction (OR 4.79, 95% CI: 1.40–16.44; I^2^ = 0%; Fig. 4). The sensitivity analysis results were consistent with the main results (Supplementary Figs. 1 and 2). In the sensitivity analysis of the studies published in English and Japanese, there was no statistically significant difference between manual and US reduction (Supplementary Figs. 3 and 4).

## Discussion

In this retrospective cohort study and systematic review, we aimed to compare the impact of non-invasive US reduction with that of manual reduction in patients with incarcerated obturator hernias. We demonstrated that non-invasive reduction had a success rate of 69%, with US reduction showing a higher success rate (78%) than manual reduction (33%). Among successful reductions, 3% required emergency surgery and bowel resections. Additionally, the majority of successful non-invasive reductions occurred within 72 h of onset. Notably, in cases in which the hernia was effectively reduced within 72 h, bowel resection was not required. This study introduces groundbreaking evidence regarding the efficacy of US-guided reduction within 72 h of symptom onset, suggesting a paradigm shift in clinical approaches to treating obturator hernias.

Our study establishes a direct link between the high success rates of non-invasive US-guided reduction techniques and our recommendation to prioritize the performance of this procedure within a vital 72-h window following symptom onset. This finding is particularly salient in the emergency medical care landscape, where the need for alternatives to immediate surgical intervention is paramount. Obturator hernias most commonly affect a vulnerable demographic—older, thin, and multiparous females—who often present with comorbidities or debilitating conditions [1]. These factors significantly reduce the safety and feasibility of emergency surgical procedures [23]. By offering a viable, non-invasive option within the crucial early intervention window, our research represents a significant advancement in the management of this condition.

The adoption of US-guided reduction techniques may dramatically improve patient outcomes by providing a safer alternative that mitigates the risks associated with conventional emergency surgeries. Supporting the critical need for early, non-invasive intervention, the existing literature reveals a significant increase in morbidity and mortality rates associated with emergency surgical treatments for complicated inguinal hernias among older adult patients [23]. This is further substantiated by data from a nationwide Danish Hernia Database cohort study, which shows a marked increase in lengths of hospital stay for patients undergoing emergency surgeries, with all observed mortalities within 30 days post-surgery occurring in this group [1]. These findings are in sharp contrast to the outcomes associated with elective repairs, which predominantly involve laparoscopic surgery and mesh repair techniques [2]. Importantly, the strategic deployment of non-invasive reduction techniques within the critical 72-h window may not only enhance the feasibility of transitioning to elective mesh repairs but may also significantly reduce the length of hospital stay, the frequency of complications, and the rate of mortality. A retrospective analysis of adult patients diagnosed with acute obturator hernias over a decade in Australia emphasized the necessity for emergency surgery in all instances [3]. This underscores not only the critical importance of the time of onset but also highlights a potential lack of awareness regarding the efficacy of noninvasive repair methods. Our research underscores the need for a paradigm shift in the management of obturator hernias and advocates the early consideration of non-invasive reduction techniques. In doing so, we aim to enhance patient outcomes by minimizing the inherent risks of emergency surgeries, thereby aligning with the best practices in emergency medicine.

**Fig. 5:**
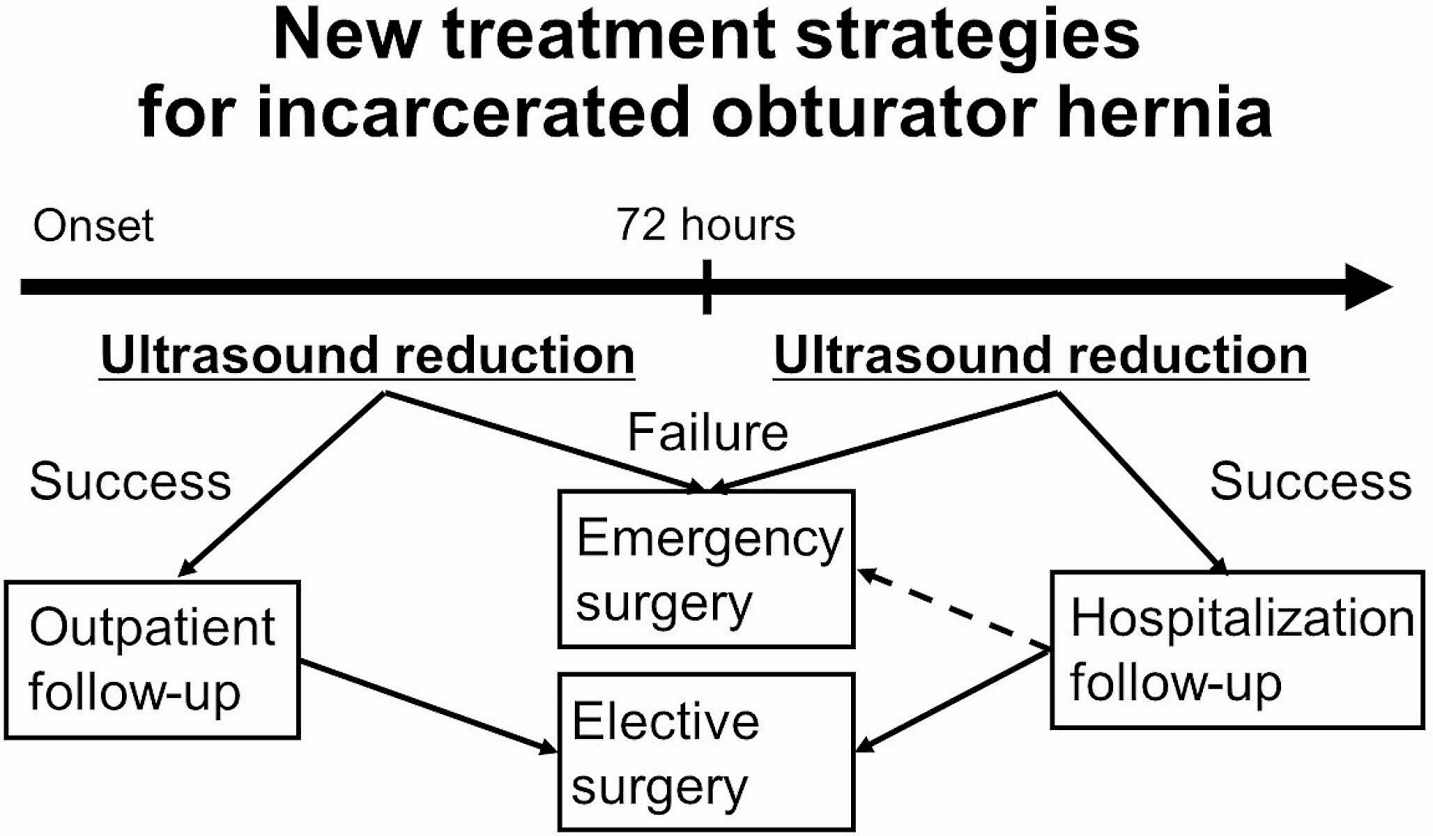
Treatment algorithm for the management of incarcerated obturator hernias

We proposed a revised treatment algorithm to optimize the management of incarcerated obturator hernias, emphasizing the importance of early intervention (Fig. 5). Ultrasound-guided reduction should be attempted in patients without any signs of intestinal necrosis. If this reduction is successful within 72 h of symptom onset, the patient may be eligible for outpatient monitoring and considered for elective surgery, allowing for a planned approach in less urgent situations. Conversely, if reduction fails within this critical timeframe, immediate surgical intervention is required. Additionally, if a successful reduction occurs after the 72-h window, inpatient monitoring is recommended to ensure thorough observation and prompt management of any potential complications. This strategic approach ensures that patients receive tailored care based on the timing and outcome of the initial non-invasive intervention, significantly enhancing safety and overall outcomes.

The principal challenge in this treatment paradigm lies in the early detection of obturator hernias, a task complicated by the often ambiguous symptomatology that closely mimics that of small bowel obstruction, including abdominal distension, pain, and vomiting [2, 24]. Compounding evidence supports the role of preoperative CT in markedly enhancing the diagnostic accuracy of obturator hernias, thereby minimizing postoperative complications and reducing the need for bowel resection as well as the mortality rate associated with these hernias [2]. The accessibility and routine application of CT imaging in Japan exemplifies the benefits of early and precise diagnosis [25]. However, the reality remains that early CT imaging may not always be feasible. In such contexts, the ability to suspect and subsequently identify obturator hernias becomes indispensable, especially among high-risk demographics such as slender, older adult women who present with non-specific symptoms, including vomiting, abdominal discomfort, and medial thigh pain [1]. Under these circumstances, US examination of the obturator foramen has emerged as a practical and effective diagnostic alternative [26, 27]. This approach not only reinforces the significance of swift and accurate diagnosis but also champions the utilization of non-invasive treatment strategies, aiming to significantly improve the prognosis for patients with obturator hernias.

In previous studies, the use of US for the reduction of incarcerated hernias did not require sedation and relaxation, but sedation and relaxation should be used in all cases in the future. Another non-invasive strategy used primarily for inguinal hernias, the “taxi” method, integrates manual reduction with the administration of analgesics or sedatives [28]. A noteworthy prospective study demonstrated that US-guided techniques could potentially reduce the requirement for emergency surgeries from approximately 10–2% [29]. Despite these advances, the application of “taxi” methods specifically for obturator hernias remains unexplored. In our analysis, the variation in success rates observed among different institutions suggests disparities in the anatomical understanding of the obturator foramen and the procedural proficiency unique to each center. To bridge these gaps, our future research will investigate the potential benefits of combining analgesic or sedative administration with US-guided reduction techniques in all cases, particularly in cases of incarcerated obturator hernias that do not resolve within the critical 72-h window. A key aspect of future work will be the vigilant monitoring of intestinal necrosis, a severe complication that underscores the urgency for optimized intervention strategies in these patients.

This study had several limitations. First, although non-invasive reduction is a relatively simple technique, the experience and expertise of the surgeon, who is also involved in the selection of each reduction technique, may affect the success rate. The accuracy of the preoperative diagnosis and patient cooperation may also have an impact. Second, this study had a relatively small sample size, and all the studies included in this review were retrospective. Further prospective observations and analyses are necessary to confirm the effectiveness of the non-invasive reduction method.

In conclusion, our study demonstrates the significant advantage of US-guided reduction as a primary, non-invasive treatment for obturator hernia within the critical 72-h window from symptom onset, showing a higher success rate and reduced need for emergency surgery compared with manual reduction. These findings underscore the importance of early, non-invasive interventions in improving patient outcomes for conditions that frequently affect vulnerable populations. However, the retrospective nature of our analysis and the variability in success rates across institutions highlight the need for future prospective studies to further validate these results and explore the potential benefits of integrating analgesic or sedative administration with US-guided reductions. Moving forward, this study could refine emergency treatment protocols and enhance the care of patients with obturator hernias.

## Electronic supplementary material

Below is the link to the electronic supplementary material.

Supplementary Material 1

## Data Availability

Our database contains highly confidential data, which may provide insight into the clinical and personal information of patients and lead to their identification. Therefore, owing to organizational restrictions and regulations, these data cannot be made publicly available. However, the datasets used and/or analyzed in the current study are available from the corresponding author upon reasonable request. In addition, this meta-analysis utilized data sourced from existing research publications, thereby making all data and study materials publicly accessible. Researchers seeking detailed individual-level data from the studies included in this meta-analysis should reach out to the corresponding author of each study for inquiries.
