# Correction to: The impact of non-invasive manual and ultrasonographic reduction for incarcerated obturator hernia: a retrospective cohort study and systematic review

**DOI:** 10.1007/s10029-024-03135-4

**Published:** 2024-08-20

**Authors:** Fuyumi Kobayashi, Jun Watanabe, Masaru Koizumi, Hironori Yamaguchi, Naohiro Sata

**Affiliations:** 1 Department of Surgery, Division of Gastroenterological, General and Transplant Surgery, Jichi Medical University, Shimotsuke-City, Tochigi Japan; 2 Department of Surgery, Tochigi Medical Center Shimotsuga, Tochigi-City, Tochigi Japan; 3 Division of Community and Family Medicine, Jichi Medical University, 3311-1 Yakushiji, Shimotsuke-City, Tochigi 329-0498 Japan

**Correction to: Hernia** 10.1007/s10029-024-03119-4

Table 1 of article “The impact of non-invasive manual and ultrasonographic reduction for incarcerated obturator hernia: a retrospective cohort study and systematic review” DOI: 10.1007/s10029-024-03119-4 reported a few errors:

Table 1:

1. For the Ultrasound-assisted reduction group:

* Duration of symptoms (hours) should be “5 [3–24]”

* Charlson comorbidity index should be “6 [5–7]”

2. For the Manual reduction group:

* Charlson comorbidity index should be “6 [6–7]”

The Publisher apologises for the mistake.

The original article has been corrected.

