# Supplementary material for: The impact of non-invasive manual and ultrasonographic reduction for incarcerated obturator hernia: a retrospective cohort study and systematic review
Source: Hernia. 2024 Jul 29;28(5):1547–57. doi: 10.1007/s10029-024-03119-4 (PMC11449992; doi:10.1007/s10029-024-03119-4)

Supplementary 1. The electronic database search strategy

CENTRAL search strategy

#1. [mh "Hernia, Obturator"]

#2. Obturator:ti,ab AND hernia*:ti,ab

#3. #1 OR #2

#4. reduc*:ti,ab OR reposition*:ti,ab OR releas*:ti,ab

#5. #3 AND #4

MEDLINE (via PubMed) search strategy

#1. " Hernia, Obturator "[Mesh]

#2. Obturator[tiab] AND hernia*[tiab]

#3. #1 OR #2

#4. reduc*[tiab] OR reposition*[tiab] OR releas*[tiab]

#5. #3 AND #4

EMBASE (via ProQuest Dialog) search strategy

S1 EMB.EXACT.EXPLODE("obturator hernia")

S2 (TI(Obturator) OR AB(Obturator)) AND (TI(hernia*) OR AB(hernia*))

S3 S1 OR S2

S4 TI(reduc*) OR AB(reduc*) OR TI(reposition*) OR AB(reposition*) OR TI(releas*) OR AB(releas*)

S5 S3 AND S4

Ichyushi Web (Japanese Medical Abstract Society database)

(閉鎖孔ヘルニア/TH or 閉鎖孔ヘルニア/AL) and ((整復/TH or 整復/AL) or 低侵襲/AL)

Supplementary 2: The trial registry search strategy

ICTRP search strategy

(Obturator AND hernia) AND (reduction OR reposition OR release)

ClinicalTrials.gov search strategy

Condition or disease: Obturator AND hernia

Supplementary 3. Summary of the characteristics of our patients with incarcerated obturator hernia

Cases	Age (year)	Sex	Lesion site	BMI (kg/m^2)	CCI Bedridden/ dementia/ CVD/ cancer	Chief complaint	Time from onset to reduction (hour)	Reduction methods	Successful reduction (Yes/No)	Emergency surgery	Surgical methods	Bowel resection cases (Yes/No)
1	89	Female	Right	18.4	6 0/0/0/0	HRS	3	Manual	Yes	No	Anterior approach	No
2	95	Female	Right	15.9	6 1/1/0/0	Vomiting, abdominal pain	6	Manual	Yes	No	Observation	–
3	87	Female	Right	15.5	6 0/0/0/0	Vomiting, low back pain	5	US-assisted	Yes	No	Anterior approach	No
4	76	Female	Right	17.8	7 0/0/1/0	Abdominal pain	5	US-assisted	Yes	Yes	Open	No
5	92	Female	Left	17.1	14 1/0/0/1	Vomiting, abdominal pain	>72	Manual	No	Yes	Open	Yes
6	85	Female	Left	17.9	7 0/0/1/0	Vomiting	72	Manual	No	Yes	Open	Yes
7	95	Female	Left	19.4	7 0/1/1/0	Vomiting	24	US-assisted	Yes	No	Observation	–
8	84	Female	Right	22.0	5	HRS	5	US-	Yes	No	Observation	–

					0/0/0/0			assisted				
9	93	Female	Right	18.2	8 0/1/1/0	Vomiting	36	US-assisted	Yes	No	Anterior approach	No
10	91	Female	Left	17.9	9 1/1/1/0	Vomiting	24	US-assisted	Yes	No	Observation	–
11	86	Female	Right	15.2	5 0/0/0/0	Vomiting	24	US-assisted	Yes	No	Anterior approach	No
12	82	Female	Right	16.2	6 1/1/0/0	Vomiting, abdominal pain	3	US-assisted	Yes	No	Laparoscopy	No

BMI, body mass index; CCI, charlson comorbidity index; HRS, Howship-Romberg sign; US, ulstrasound

Supplementary 4. Risk of bias for the eligibility studies

Authors [ref no.]	ROBINS-I assessment							
	Bias due to confounding	Bias in selection of participants into the study	Bias in classification of interventions	Bias due to deviations from intended interventions	Bias due to missing outcome data	Bias in measurement of the outcome	Bias in selection of the reported results	Overall risk of bias
Shigemitsu [7]	Critical	Moderate	Serious	High	Moderate	Low	No information	Critical
Mikami [18]	Critical	Moderate	Low	High	Moderate	Low	No information	Critical
Tonouchi [19]	Critical	Serious	Serious	High	Critical	Low	No information	Critical
Kawanaka [9]	Critical	Moderate	Moderate	High	Moderate	Low	No information	Critical
Hara [20]	Critical	Moderate	Low	High	Moderate	Low	No information	Critical
Maeda [21]	Critical	Moderate	Moderate	High	Moderate	Low	No information	Critical
Togawa [8]	Critical	Low	Moderate	High	Moderate	Low	No information	Critical
Gokon [6]	Critical	Moderate	Moderate	High	Moderate	Low	No information	Critical
Fujii [22]	Critical	Low	Low	High	Moderate	Low	No information	Critical

The risk of bias using Risk of Bias 2; Low: Risk of bias was low. Some concerns; Risk of bias was some concerns. High; Risk of bias was high.

Supplementary figure 1. Forrest plot of success rate between manual and ultrasound reduction in inclusion of studies assuming that the next case fails in a study with a 100% success rate

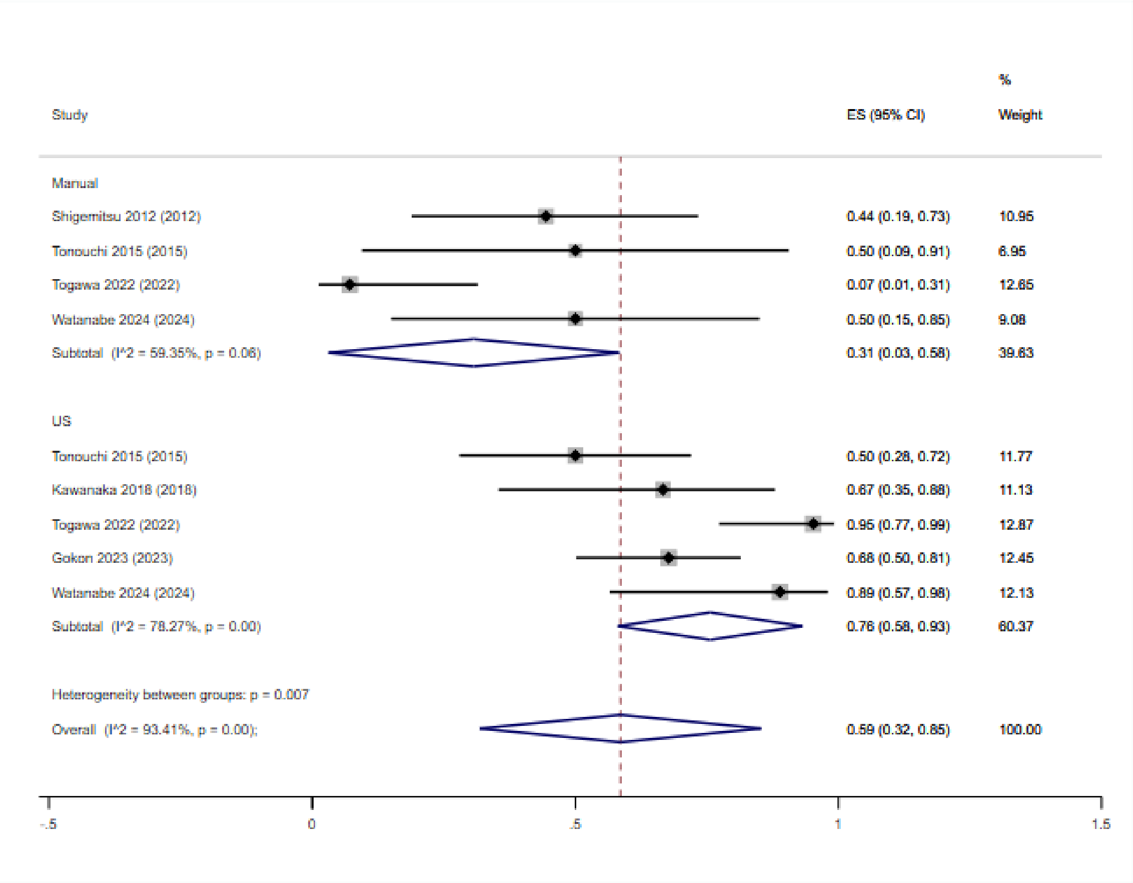

Supplementary figure 2. Forrest plot of success rate between manual and ultrasound reduction in exclusion of studies with 100% success rate

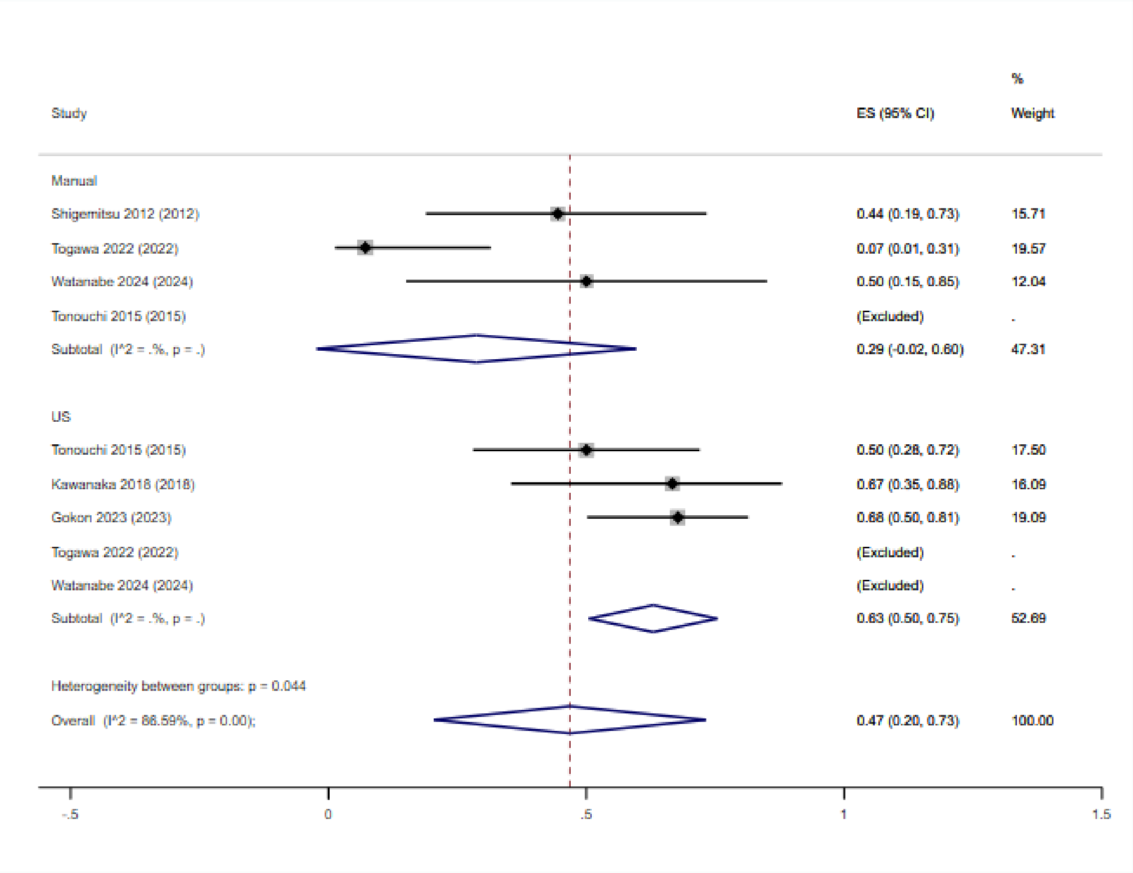

Supplementary figure 3. Forrest plot of success rate between manual and ultrasound reduction in inclusion of studies assuming that the next case fails in a study with a 100% success rate in studies published in English and Japanese

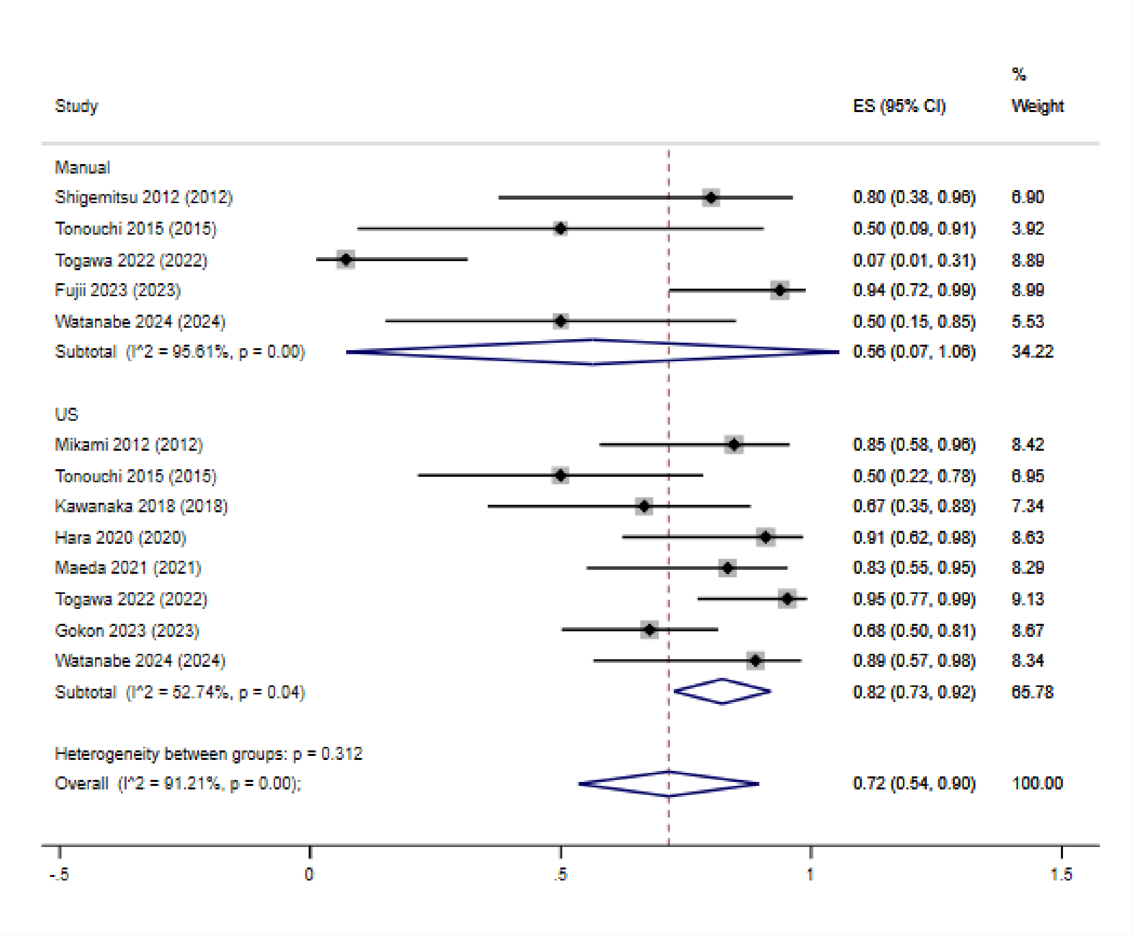

Supplementary figure 4. Forrest plot of success rate between manual and ultrasound reduction in exclusion of studies with 100% success rate in studies published in English and Japanese

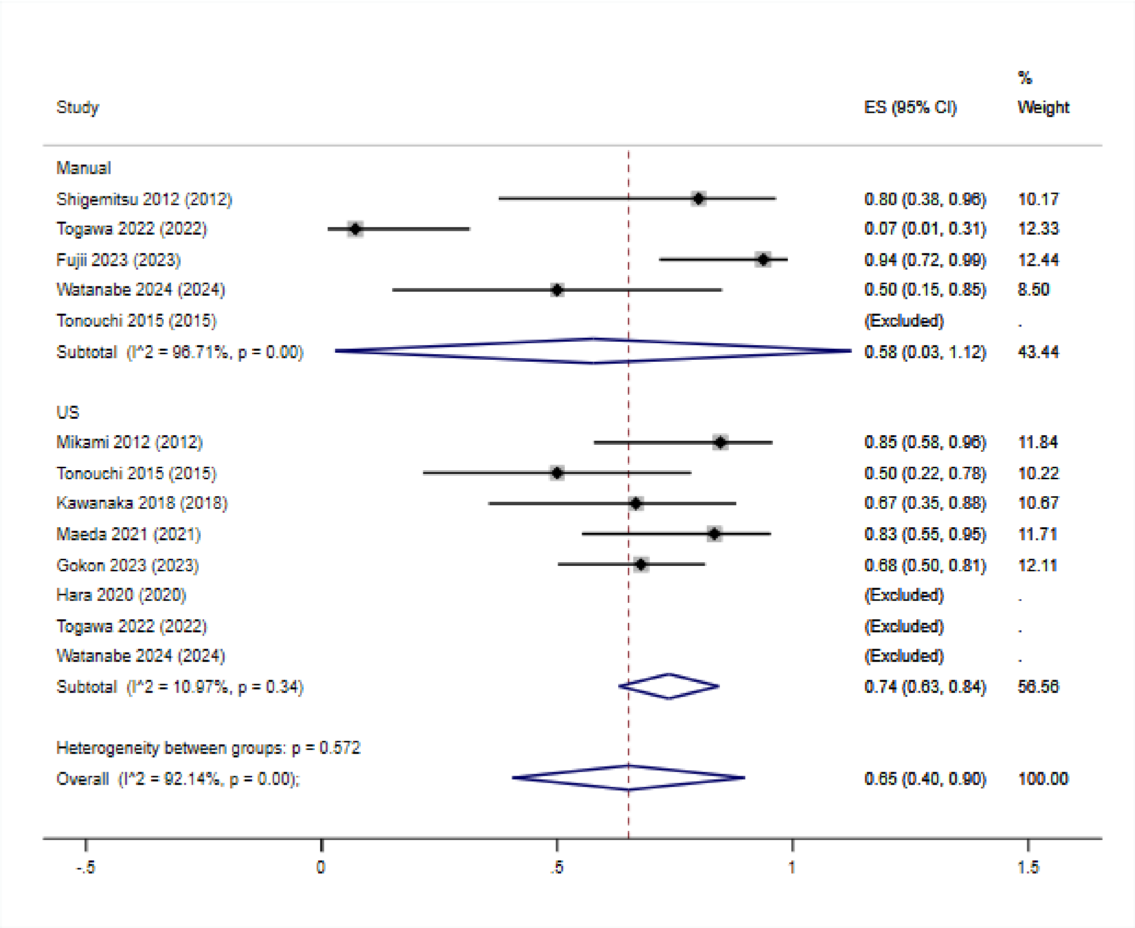

Supplement: Supplementary file 1 — Supplementary Material 1 [file 10029_2024_3119_MOESM1_ESM.pdf]
